# Antitumor Activities of Kushen: Literature Review

**DOI:** 10.1155/2012/373219

**Published:** 2012-08-28

**Authors:** Mingyu Sun, Hongyan Cao, Lin Sun, Shu Dong, Yanqin Bian, Jun Han, Lijun Zhang, Shuang Ren, Yiyang Hu, Chenghai Liu, Lieming Xu, Ping Liu

**Affiliations:** ^1^Key Laboratory of Liver and Kidney Diseases, Institute of Liver Diseases, Shuguang Hospital, Shanghai University of Traditional Chinese Medicine, 528 Zhangheng Road, Pudong New Area, Shanghai 201203, China; ^2^Shanghai University of Traditional Chinese Medicine, 528 Zhangheng Road, Pudong New Area, Shanghai 201203, China; ^3^E-institute of Shanghai Municipal Education Commission, Shanghai 201203, China; ^4^Liaoning University of Traditional Chinese Medicine, Shenyang 100032, China; ^5^School of Pharmacy, Second Military Medical University, Shanghai 201203, China

## Abstract

To discover and develop novel natural compounds with therapeutic selectivity or that can preferentially kill cancer cells without significant toxicity to normal cells is an important area in cancer chemotherapy. Kushen, the dried roots of *Sophora flavescens* Aiton, has a long history of use in traditional Chinese medicine to treat inflammatory diseases and cancer. Kushen alkaloids (KS-As) and kushen flavonoids (KS-Fs) are well-characterized components in kushen. KS-As containing oxymatrine, matrine, and total alkaloids have been developed in China as anticancer drugs. More potent antitumor activities were identified in KS-Fs than in KS-As *in vitro* and *in vivo*. KS-Fs may be developed as novel antitumor agents.

## 1. Introduction

To discover and develop novel natural compounds with therapeutic selectivity or that can preferentially kill cancer cells without significant toxicity to normal cells is an important area in cancer chemotherapy. Because of their wide range of biological activities and low toxicity in animal models, some natural products have been used as alternative treatments for cancers. Many anticancer drugs are derived from naturally occurring compounds. Vinca alkaloids (e.g., vinblastine, vincristine) and taxol are examples of such compounds.

The traditional Chinese medicine kushen is the dried roots of *Sophora flavescens *Aiton (Leguminosae). It was first described in the Chinese book *Shen Nong Ben Cao Jing* in 200 A.D. as a treatment for solid tumors, inflammation, and other diseases [1]. The traditional use of kushen includes the decoction or powder of dried plant roots. It is commonly used for the treatment of viral hepatitis, cancer, enteritis, viral myocarditis, arrhythmia, and skin diseases (e.g., colpitis, psoriasis, eczema) [2].

The known chemical components of kushen include alkaloids (3.3%), flavonoids (1.5%), alkylxanthones, quinones, triterpene glycosides, fatty acids, and essential oils [2, 3]. Kushen alkaloids (KS-As) and kushen flavonoids (KS-Fs) are well-characterized components in kushen. KS-As have been developed as anticancer drugs in China. More potent antitumor activities have been identified in KS-Fs than in KS-As [4].

## 2. KS-As 

KS-As have been well studied and are considered to be the major active components of kushen as demonstrated in experimental animal models [5–8] and clinical studies [9–14]. The bioactivities of kushen (including antitumor, anti-viral and anti-inflammatory activities) have been shown in the KS-As fraction [6].

**Figure 1:**
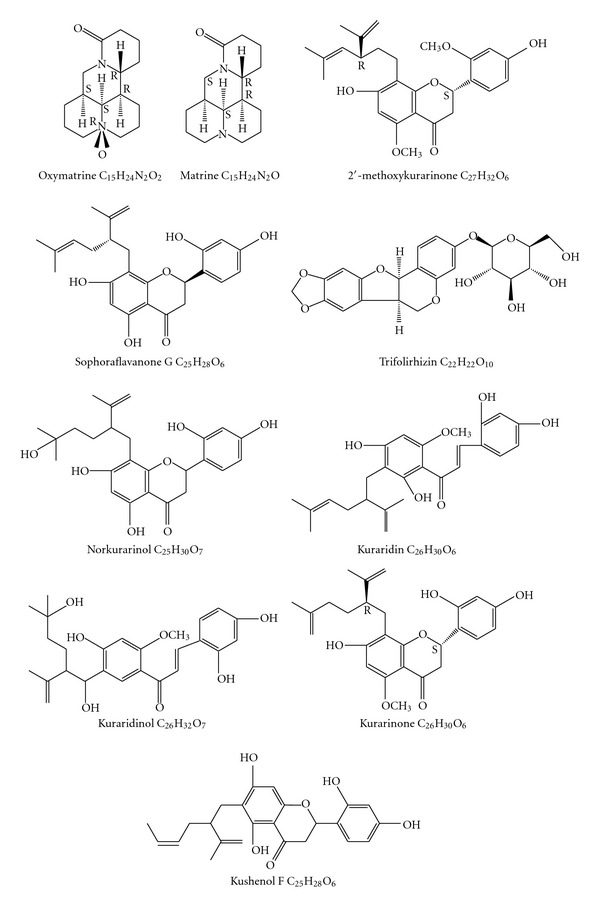
The molecular structure of antitumer compounds derived from *Sophora flavescens*.

KS-As containing oxymatrine, matrine (Figure 1), and total alkaloids were approved for the treatment of cancer patients by the Chinese State Food and Drug Administration (SFDA) in 1992. Multiple KS-As products have been used widely in China for the treatment of cancers and hepatitis. The SFDA-approved KS drugs for oncology are all KS-As used as single agents or in combination with chemotherapy or radiotherapy. Few studies focused on the efficacy of KS-As in animal models and clinical trials before 1992, when KS-As was first approved. 

 Several clinical studies reported that KS-As were efficacious in the treatment of various types of solid tumors (including lung, liver, and gastrointestinal tract). The treatment responses were comparable to, or better than, that of chemotherapy drug-treated patients (Table 1) [11, 14–32]. KS-As demonstrates a good safety profile in cancer patients, such as reduced toxicity in the bone marrow when used in combination with chemotherapy agents [33]. Long-term survival data for KS-As-treated cancer patients remains to be demonstrated with well-controlled clinical studies and large patient cohorts. 

## 3. Matrine and Oxymatrine

Matrine and oxymatrine (Figure 1) are the two major alkaloid components found in the roots of Sophora species. They are obtained primarily from *Sophora japonica* (kushen), *Sophora subprostrata* (shandougen), and from the overground portion of *Sophora alopecuroides*. The matrines were first isolated and identified in 1958; they are unique tetracyclo-quinolizindine alkaloids found only in *Sophora* species thus far [52–56].

*In vitro* studies have demonstrated that matrine and oxymatrine weakly inhibit the growth of various human tumor cell lines with a half-maximal inhibitory concentration (IC_50_) of 1.0–4.0 mg/mL [57–61]. 

*In vivo* studies have shown that KS-As, oxymatrine, and matrine inhibit the growth of murine tumors, including H22, hepatoma, S180, sarcoma, and MA737 breast cancer cells [58, 60, 62, 63]. In a human xenograft tumor model using the SGC-7901 cell line, matrine enhanced the inhibition of 5-fluorouracil in the tumor [33].

Matrine can also inhibit the invasiveness and metastasis of the human malignant melanoma cell line A375 and cervical cancer HeLa cells, as well as induce differentiation of leukemia K-562 cells [64–66]. In addition, matrine-induced autophagy in rat C6 glioma cells has been observed by electron microscopy [67].

The antitumor response of KS-As was further demonstrated in several clinical studies in various types of cancers, including stomach, esophagus, liver, colon, lung, cervix, ovary, and breast cancers, as a single agent [9–14] or in combination with chemotherapy [15–18] or radiotherapy [68]. It has been reported that matrine exerts its antitumor effects by inhibiting the proliferation and inducing the apoptosis of gastric and cervical cancer cells as well as leukemic and glioma cells [34, 67–70]. 

Several *in vitro* and *in vivo* studies have tried to elucidate the mechanism of action of matrine. Matrine promotes apoptosis in leukemic [35], breast cancer [36], nonsmall-cell lung cancer [37], hepatocarcinoma, and gastric cancer cells [38] by a mitochondrial-mediated pathway [39]. Beclin 1 is involved in matrine-induced autophagy, and the pro-apoptotic mechanism of matrine may be related to its upregulation of Bax expression [39]. Recent evidence indicates that matrine also has appreciable effects in modulating the immune response by reducing the invasion and metastasis of HCC cells [40, 41, 71]. 

Tissue homeostasis requires a balance between the division, differentiation and death of cells. A tumor is a type of “cell cycle disorder” that has the abnormal interface of division, differentiation and death [42]. As a “biological modifier” of cells, matrine can reverse the abnormal biologic behavior of tumor cells and recover the balance between the division, differentiation, and death of cells.

Matrine can also inhibit the invasiveness and metastasis of the human malignant melanoma cell line A375 [43]. Some studies reported that matrine reduced the adhesion and migration of HeLa cells [72]. The mechanisms of action of matrine against cancer cell proliferation and invasion are associated with epidermal growth factorvascular endothelial growth factor vascular endothelial growth factor receptor 1 Akt–nuclear factor-kappa B (EGF/VEGF—VEGFR1—Akt—NF-*κ*B) signaling [36] (Table 2).

 Matrine displays synergistic effects with the anticancer agents celecoxib (cyclooxygenase-2 inhibitor), trichostatin A (histone deacetylase inhibitor) and rosiglitazone against the tumor proliferation and VEGF secretion. Matrine may have broad therapeutic and/or adjuvant therapeutic applications in the treatment of human nonsmall-cell lung cancer, breast cancer, and hepatoma [36, 37] (Table 2). 

Some studies have also reported upon the anticancer activity of oxymatrine in human gastric cancer cells, pancreatic cancer, and human breast cancer cells [73–75]. Oxymatrine can induce the apoptosis death of human pancreatic cancer cells, which might be attributed to the regulation of Bcl-2 and IAP families, release of mitochondrial cytochrome C, and activation of caspase-3 [74] (Table 2). 

 Compound kushen injection (CKI), commonly known as Yanshu injection, is extracted from two herbs, kushen (Radix Sophorae Flavescentis) and Baituling (Rhizoma Smilacis Glabrae), with the primary components being oxymatrine and matrine [75]. CKI has been used extensively alone or in combination with chemotherapy or radiotherapy for many years in China. Clinical studies have shown that CKI attenuates the side effects of chemotherapy and radiotherapy by improving the quality of life and regulating the immune function of cancer patients, as well as synergizing the therapeutic effects of chemotherapy and radiotherapy (Table 1) [15–32, 76]. It has been demonstrated that CKI suppresses the growth of tumor cells by inducing apoptosis and inhibiting the migration, invasion, and adhesion, of such cells [77].

Cancer stem cells (CSCs) play an important part in the initiation, relapse and metastasis of cancer. A specific agent has not been found to target CSCs because they are resistant to most conventional therapies and proliferate indefinitely. In one study, CKI suppressed the size of the side population (SP; ~90%) and downregulated the main genes of the Wnt signaling pathway in MCF-7 SP cells. CKI suppressed tumor growth by downregulating the Wnt/b-catenin pathway, whereas cisplatin activated the Wnt/b-catenin pathway and could spare SP cells. These data suggested that CKI may serve as a novel drug targeting CSCs, but further studies are recommended [44]. 

## 4. KS-Fs 

The antitumor effects of some flavonoid compounds (Figure 1) have been demonstrated *in vitro* and *in vivo* [78–82]. Surprisingly, the antitumor activities of KS-Fs were more potent than those of KS-As, which have been considered to be the major active components in the plant [83]. KS-Fs such as kurarinone, 2′-methoxykurarinone, and sophoraflavanone G (lavandulyl flavanones isolated from *S. flavescens*) (Figure 1) can inhibit cell proliferation in A549, NCI-H460 (nonsmall-cell lung), SK-OV-3 (ovary), SK-MEL-2 (skin), XF498 (central nerve system), HCT-15 (colon) HL-60 (myeloid leukemia) and SPC-A-1 (lung) cells with IC_50_ values between 2 *μ*g/mL and 36 *μ*g/mL [80, 82, 83]. 

Antitumor efficacies were confirmed in mice models of H22, S180 and Lewis lung tumors as well as nude mice models of human H460 and Eca-109 xenografted tumors [45, 83]. Moreover, KS-Fs and kurarinone enhanced the antitumor activities of Taxol *in vitro* and *in vivo *[83, 84]. The oral or intravenous maximum tolerated dose of KS-Fs was >2.8 g/kg or 750 mg/kg, respectively, appreciably more than the oral median lethal dose of KS-As (≤1.18 g/kg). Adverse reactions were not observed. In addition, peripheral blood cell counts were not significantly affected in normal mice treated with KS-Fs at 200 mg/kg/day for 2 weeks [45, 83]. 

Kuraridin, sophora flavanone G, kurarinone, kushenol F, and norkurarinol have extremely strong tyrosinase inhibitory activity [45–49]. Kurarinol, kuraridinol, and trifolirhizin markedly inhibited (>50%) melanin synthesis [48, 49]. 

The mechanism of action of KS-Fs and kurarinone involves inhibition of tumor necrosis alpha one (TNF*α*l)-induced NF-*κ*B activation and enhance apoptosis [45, 50, 51]. The apoptosis-inducing effect was enhanced in the presence of taxol. In H460 xenograft mice treated with kurarinone, downregulation of Bcl-2 and upregulation of caspase 8 and caspase 3 in tumors were observed [45]. KS-Fs and kurarinone induce apoptosis in tumors by acting on multiple cellular targets, including inhibition of NF-*κ*B activation and multiple receptor tyrosine kinase activities [45]. Kurarinone and kuraridin attenuate NF-*κ*B activation by inhibition of I*κ*B*α* proteolysis and p65 nuclear translocation, as well as phosphorylation of extracellular signal-regulated kinase (ERK)1/2, c-Jun N-terminal kinase (JNK), and p38 mitogen-activated protein kinases [45, 51]. Constitutive NF-*κ*B and RSK2 activities are important hallmarks of human cancers (including hematopoietic malignancies and solid tumors), so prenylated flavanones represent an attractive class of natural inhibitors of the ERK/RSK2 signaling pathway for cancer therapy [85] (Table 2).

Fifty-six flavonoids have been identified from KS-Fs. Twenty-one of the KS-Fs have been found to have antitumor activities. Studies have demonstrated that more potent antitumor activities are observed in KS-Fs instead of KS-As fractions. KS-Fs were more than 10-fold more potent than KS-As in the cell proliferation assay. Further evaluation of the safety and efficacy of KS-Fs in clinical oncology settings is warranted. KS-Fs could be developed as botanical drugs for solid tumors, and kurarinone could be used as a marker compound. Additional structural modifications of KS-Fs compounds could also generate more potent drug candidates. 

## 5. Conclusions and Future Perspectives

This paper summarized the antitumor efficacy and mechanism of action of kushen and its constituents *in vitro* and *in vivo*. Many Patents of kushen extracts have been applied in USA, China and other countries (Table 3). These results strengthen the hypothesis that kushen (or its components) alone or combination with chemotherapy agents could modulate various molecular pathways in tumors or be used to treat cancer. Studies described here and elsewhere highlight the use of flavonoids of kushen as novel chemoprevention agents for cancer intervention. It is expected that future studies with kushen will help to define various molecular mechanisms and targets for the inhibition and apoptosis of tumor cells. The number of multicenter, large sample, randomized, double-blind, controlled chemoprevention clinical trials with kushen are very limited. Extensive clinical research is warranted to evaluate further the safety and chemoprevention efficacy of kushen alone or in combination with chemotherapy agents.

## Figures and Tables

**Table 1:** Clinical trials using compound kushen injection.

	Dose and course of treatment	Combined medication	Case/control	Cancer type	Indications and symptoms	Efficacy	Positive control	Side effect	Reference
1	1,000–1,500 mg + GS 500 mL, i.v., q.d., 30–45 days	No	68/37	Gastric cancer	Fever, pain, GI reactions, ascites	Relief	MMC + UFT	Abdominal distention, constipation	[11]

2	1,000–1,500 mg + GS 500 mL, i.v., q.d., 30 days, total dose of 30–45 g	No	44	Hepatocarcinoma	Evaluation of curative effect, immune effect, toxic effect	Effective treatment, reduction in tumor size, improvement in symptoms and signs, improvement in immune function	No	Nausea	[14]

3	30 mL + GS 250 mL, i.v., q.d., 10 days	Hydroxycamptothecin	20/20	Hepatocellular carcinoma	Recurrence rate	HCPT and CKI postoperative arterial infusion may be helpful for reducing intrahepatic recurrence after curative resection for HCC	PDD and 5-FU	No	[15]

4	1,000 mg + GS 500 mL, i.v., q.d., 30 days	Carboplatin or 5-FU	21	Malignant ascites	Evaluation of curative effect (ascites)	Lessening of ascites	Carboplatin or 5-FU	Abdominal distention	[16]

5	50 mL intrapleural injection or 100 mL peritoneal injection, b.i.w., 3 weeks	Dexamethasone	24	Virulent succus inside the thorax and belly: lung cancer, breast cancer, ovarian cancer	Ascites	Lessening of ascites, effective rate (CR and PR) 68.8%	No	Mild abdominal pain	[17]

6	1,000 mg + GS 500 mL, i.v., q.d., 30 days, 2–4 cycles	MVP/FAM/CAF/FP	65/61	Malignant tumor: lung, esophageal, liver, gastric, breast, colon, nasopharyngeal	Toxic effect, QoL	Improvement in QoL, reduce the toxic effects (leukopenia, GI reactions) of chemotherapy	MVP/FAM/CAF/FP	No	[18]

7	12–15 mL + NS 250 mL, i.v., q.d., 10 days	No	52/52	Malignant tumor: lung, breast, gastric, esophageal, colorectal, pancreatic	Pain	Pain relief	Sustained-release indomethacin or tramadol hydrochloride	No	[19]

8	15 mL + NaCl 250 mL, i.v., q.d., for 12 days	Fentanyl	31/31	Advanced cancer: gastric, esophageal, breast	Pain	Pain relief and KPS better than fentanyl, *P* < 0.05	Fentanyl alone	No	[20]

9	20 mL + NaCl 250 mL, i.v., q.d., for 14 days, 3 cycles	Oxaliplatin and capecitabine	36/30	Advanced gastric cancer in senile subjects	KPS; toxic effect	KPS better than oxaliplatin and capecitabine, *P* < 0.05; CKI has a low incidence rate for leukopenia, pain and hepatotoxicity, *P* < 0.05	Oxaliplatin and capecitabine	No	[21]

10	20 mL, i.v., q.d., 10–14 days	Chemotherapy: CAVP or CAP	61/60	Lung cancer	Immune	Improvement in immune function	Chemotherapy: CAVP or CAP	No	[22]

11	30 mL + NaCl 250 mL, i.v., q.d., 7 days	Taxol and epirubicin	34/34	Breast cancer	Immune	Improvement in immune function	Taxol and epirubicin	No	[23]

12	20 mL + GS/NaCl 250 mL, i.v., q.d., 14 days, 2 cycles	TACE (5-FU 1,000–2,000 mg, MMC 10–20 mg, EP I60–100 mg)	27/23	Liver cancer	KPS; toxic effect	KPS better than TACE, *P* < 0.05; The incidence rate of nausea and vomiting hepatotoxicity were significantly lower than for TACE chemotherapy, *P* < 0.05	TACE	No	[24]

13	30 mL + GS/NaCl 250 mL, i.v., q.d., 10 days, 4 cycles	FOLRIRI chemotherapy	50/50	Advanced colorectal cancer	Pain, toxic effect, KPS	Pain relief, reduced toxic effect (leukopenia, GI reactions, hepatotoxicity and renal toxicity) of FOLFIRI chemotherapy; improved QoL	FOLRIRI chemotherapy	No	[25]

14	20 mL, i.v., q.d., 14 days, 3 cycles	Radiotherapy	33/33	Cervical cancer	Evaluation of curative effect, KPS, immune toxic effect	Improve therapeutic effects, QoL, immune function; attenuate myelosuppression	Radiotherapy alone	No	[26]

15	10 mL + NaCl 250 mL i.v. q.d. 10 days	DA/TA/MA chemotherapy	35/35	AML	KPS, hematotoxicity	Improve QoL; attenuate hematotoxicity (leukopenia) of chemotherapy	DA/TA/MA chemotherapy	No	[27]

16	20 mL intrapleural injection; keep 48 h q2W and 20 mL + 100 mL NaCl, i.v., q.d., 4 weeks	No	32/32	Malignant pleural effusion	KPS, evaluation of curative effect, toxic effect	Improve QoL and therapeutic effects; reduce toxic effects	Cisplatin	No	[28]

17	100 mL, i.v. b.i.d., 10 days	Gastric cancer: TPF chemotherapy; colorectal cancer: FOLFOX or FOLFRI chemotherapy; breast cancer: TA or CAF chemotherapy; lung cancer: GP/TP/NP chemotherapy	83/83	Malignant tumors: gastric, lung, colorectal, breast	Hepatotoxicity	CKI can effectively prevent hepatic injury caused by chemotherapy (incidence and degree of hepatic injuries)	Chemotherapy: gastric cancer: TPF; colorectal cancer FOLFOX or FOLFRI; breast cancer: TA or CAF; lung cancer GP/TP/NP	No	[29]

18	20 mL + NaCl 250 mL, i.v., q.d., 21 days, 2 cycles	FOLFOX4 chemotherapy	27/21	Gastric cancer	Toxic effect	Incidence rate of alopecia lower than for FOLFOX4 chemotherapy, *P* < 0.05.	FOLFOX4 chemotherapy	No	[30]

19	20 mL + NaCl 200 mL, i.v., q.d., 14 days, 2 cycles	FOLFX chemotherapy	30/30	Gastric cancer	Toxic effect, QoL, symptoms	Promote reduction of symptoms, reduce chemotherapy side effects (alopecia, leukopenia, thrombocytopenia, GI reactions), improve QoL and prolong median survival time	FOLFX chemotherapy	No	[31]

20	20 mL in NaCl 250 mL, i.v., q.d., for 3-4 weeks, 2-3 cycles	Chemotherapy and radiotherapy	75/75	Mid-late-stage cancer: lung, breast, esophageal, nasopharyngeal, colorectal, pancreatic, ovarian	Immune, CBR, KPS, toxic effect	Improvement in immune function, increase the CBR and QoL and reduce adverse reactions of chemotherapy in patients with midlate-stage cancer.	Chemotherapy and radiotherapy (lung cancer: NP/GP/TP + radiotherapy; breast cancer: CAF/TA + radiotherapy: esophageal cancer: PF + radiotherapy; nasopharyngeal carcinoma: DDP + radiotherapy; colorectal cancer: oxaliplatin + 5FU + CF/oxaliplatin + xeloda; pancreatic cancer: GP; ovarian cancer: CAP/TP)	No	[32]

AML: acute myeloid leukemia; CAF: cyclophosphamide, adriamycin, and fluorouracil; CAP: cyclophosphamide, doxorubicin, and cisplatin; CAVP: cyclophosphamide, doxorubicin, and etoposide; CBR: clinical benefit rate; CF: calcium 5-formyletrahydrofolate; CR: complete remission; DA: daunorubicin and cytarabine; DDP: cisplatin; FAM: fluorouracil, adriamycin, and mitomycin; FOLFOX: oxaliplatin, calcium folinate, and fluorouracil; FOLFOX4: oxaliplatin, calcium folinate and fluorouracil; fOLFRI: irinotecan, calcium folinate and fluorouracil; FOLFX: oxaliplatin, calcium folinate and fluorouracil; FOLRIRI: leucovorin, fluorouracil, and irinotecan; FP: fluorouracil and cisplatin; 5-FU: fluorouracil; GI: gastrointestinal; GP: gemcitabine and cisplatin; HCC: hepatocellular carcinoma; HCPT: hydroxycamptothecin; KPS: karnofsky performance scale; MA: mitoxantrone and cytarabine; MMC: mitomycin; MVP: mitomycin, vinblastine, and cisplatin; NP: vinorelbine and cisplatin; PDD: cisplatin; PR: partial remission; QoL: quality-of-life; TA: paclitaxel and epirubicin; TA(9): pirarubicin and cytarabine; TACE: fluorouracil, mitomycin, and epirubicin; TP: paclitaxel and cisplatin; TPF: paclitaxel, fluorouracil, and cisplatin; UFT: Tegafur-Uracil.

**Table 2:** Mechanism of action of the chemotherapy of kushen compounds.

Compound	Mechanisms of action	Reference
Matrine	Promotes apoptosis via mitochondria	[34–38]
	Modulates the immune response	[39–41]
	Inhibits EGF/VEGF—VEGFR1—Akt—NF-*κ*B signaling	[35, 42, 43]
Compound kushen injection	Inhibits cancer stem cells	[44]
Kuraridin, sophoraflavanone G, kurarinone, kushenol F, and norkurarinol	Strong tyrosine kinase inhibitory activity	[45–49]
Kurarinone	Inhibits TNF*α*l-induced NF-*κ*B activation and enhances apoptosis	[45, 50, 51]
Kurarinone and kuraridin	Attenuate NF-*κ*B activation by inhibition of I*κ*B*α* proteolysis and p65 nuclear translocation as well as phosphorylation of ERK1/2, JNK, and p38 MAP kinases	[45, 51]

**Table 3:** Patents of kushen extracts.

Patent	Patent number
Extract of *Sophora flavescens* flavonoids and uses thereof	US20050226943
Prenylated flavonoid derivatives having anti-inflammatory properties and *Sophora flavescens* extracts	Korea1020000077932
Extract of *Sophora flavescens* flavonoids and uses thereof	US20050226943
Compositions comprising matrine and dictamnine for treating or preventing cancer and other diseases	US2004192579A1
Medicine preparation containing matrine or epimatrine and its application in analgesic medicines	CN1347694A
Use of oxidized matrine in preparation of chemicals for treating venereal diseases	CN1530108A
A process for the manufacture of a herbal composition comprising a matrine	WO02067955A3
Use of oxidized matrine in preparation of chemicals for treating viral myocarditis	CN1530109A
Double salt formed by inosine and matrine or oxymatrine and application thereof in field of medications	CN101724002A
Pharmaceutical purpose of compound comprising ferulic acid and matrine alkaloid in prevention and treatment of osteoarthropathy	CN101669946A
Application of oxidized matrine in preparing medicine for treatment of hepatitis B	CN1157717A
Joint synergy of ferulic acid and matrine alkaloid and medical application thereof	CN101669945A
Medicinal use of matrine alkaloid for promoting digestive tract power	CN1850075A
Medicine composition containing silymarin and kurarinone or matrine and use thereof	CN101357129A
Pharmaceutical composition comprising kurarinone, magnolia vine fruit, and ginseng for treating hepatitis	CN1970001A
Use of kurarinone in preparation of medicine for postoperative intestine functional restoration	CN1923198A
Combination of medication of containing kurarinone and glycyrrhetic acid, and application	CN1695624A
Oxymatrine compositions and related methods for treating and preventing chronic infectious diseases	US2010022575A1
Pharmaceutical composition comprising oxymatrine and baicalin	CN1919205A
Medicinal composition of oxymatrine and polysaccharide	CN101081240A
Complex salt of silybin and oxymatrine or matrine and uses thereof	CN101157689A
Double salt formed by inosine and matrine or oxymatrine and application thereof in field of medications	CN101724002A
Method for separating matrine and oxymatrine from total matrines	CN101585837A
Application of oxymatrine in preparing medicine for treating acute chronic cardiac insufficiency disease	CN101185647A
Application of oxymatrine in preparing medicine to treat viral hepatitis C	CN1350848A
Application of oxymatrine in preparing medicine to treat liver fibrosis	CN1350849A
Use of alkaloids extracted from *Sophora flavescens* in preparing medicine for treating diseases reduced by mycoplasma, chlamydia and fungus	CN101336958A
Compositions for improving skin conditions comprising matrine or its oxidized derivatives	US2010099698A1
Oxymatrine compositions and use thereof for treating and preventing chronic infectious diseases	WO2010011975A1
Preparation and use of silybin bis bias succinate oxymatrine double salt and matrine double salt	CN101297802A
Medication with spermicidal effect *in vitro* and bacteriostatic action and preparation method and application thereof	CN101757140A
Chinese medicine for hepatitis B and its preparation	CN1244409A
Medicinal composition for preventing tumors	CN101073611A
Application of kushen (*Sophora flavescens*) flavone in preparing antihypoglycemic agents	CN1348762A
